# Japan Unified Protocol Clinical Trial for Depressive and Anxiety Disorders (JUNP study): study protocol for a randomized controlled trial

**DOI:** 10.1186/s12888-016-0779-8

**Published:** 2016-03-18

**Authors:** Masaya Ito, Yasuyuki Okumura, Masaru Horikoshi, Noriko Kato, Yuki Oe, Mitsuhiro Miyamae, Naotsugu Hirabayashi, Ayako Kanie, Atsuo Nakagawa, Yutaka Ono

**Affiliations:** National Center for Cognitive Behavior Therapy and Research, National Center of Neurology and Psychiatry, Ogawa Higashi 4-1-1, Kodaira, Tokyo 187-8511 Japan; Institute for Health Economics and Policy, Association for Health Economics Research and Social Insurance and Welfare, Tokyo, Japan; National Center of Neurology and Psychiatry, Tokyo, Japan; Center for Clinical Research, Keio University School of Medicine, Tokyo, Japan; Center for the Development of Cognitive Behavior Therapy Training, Tokyo, Japan

**Keywords:** Anxiety disorder, Depressive disorder, Emotion, Transdiagnostic, Unified protocol, Randomized controlled trial

## Abstract

**Background:**

The unified protocol for the transdiagnostic treatment of emotional disorders is a promising treatment approach that could be applicable to a broad range of mental disorders, including depressive, anxiety, trauma-related, and obsessive-compulsive disorders. However, no randomized controlled trial has been conducted to verify the efficacy of the unified protocol on the heterogeneous clinical population with depressive and anxiety disorders.

**Methods/design:**

The trial was designed as a single-center, assessor-blinded, randomized, 20-week, parallel-group superiority study in order to compare the efficacy of the combination of unified protocol and treatment-as-usual versus waiting-list with treatment-as-usual for patients with depressive and/or anxiety disorders. The primary outcome was depression at 21 weeks, assessed by the 17-item version of the GRID-Hamilton Rating Scale for Depression. Estimated minimum sample size was 27 participants in each group. We will also examine the treatment mechanisms, treatment processes, and neuropsychological correlates.

**Discussion:**

The results of this study will clarify the efficacy of the unified protocol for depressive and anxiety disorders, and the treatment mechanism, process, and neurological correlates for the effectiveness of the unified protocol. If its efficacy can be confirmed, the unified protocol may be of high clinical value for Japan, a country in which cognitive behavioral treatment has not yet been widely adopted.

**Trial Registration:**

ClinicalTrials.gov NCT02003261 (registered on December 2, 2013)

## Background

### Transdiagnostic treatment for emotional disorders

Emotion is one of the biggest sources of human distress. Symptoms of emotional difficulties, in particular depression and anxiety, are among the most common symptoms observed in psychiatric and clinical-psychological settings. Lifetime prevalence of depressive and anxiety disorders is estimated at 6.3 and 6.7 % in Japan [1], and 16.6 and 28.8 % each in the U.S. [2]. Depressive and anxiety disorders were respectively ranked as the second- and ninth-largest causes of global years lived with disability in 2013 [3], and depressive disorders in particular are predicted to become the first cause of disease burden by 2030 [4]. The economic burden of depressive disorder has been enormous ($11–18 billion in Japan and $173–211 billion in the U.S. in 2010) [5–7]. Similarly, the economic burden of anxiety disorders was reported as $20.5 billion in Japan and $42.3 billion in the U.S. in 1990 [8, 9]; and it should be noted that this figure for anxiety disorders in the U.S. could be greatly underestimated, because it did not consider long-term opportunity cost or the cost associated with comorbidity, which could bring total costs to the U.S. economy as a result of anxiety disorder to an estimated $100 billion [10].

Depressive and anxiety disorders, which are commonly comorbid, are both conceptualized as disorders of emotion [11]. Accumulated findings show shared psychopathology, etiology, neurobiological characteristics, and similar cognitive-affective, interpersonal, and behavioral-maintenance factors commonly observed among depression, anxiety, and trauma-related, obsessive-compulsive, and other emotion-related disorders [11–15]. These findings leads to the idea of transdiagnostic conceptualization of these disorders [16]; the term often used is “emotional disorders” [15]. This broad category includes, at minimum, the *Diagnostic and Statistical Manual of Mental Disorders* 5th edition (DSM-5) categories of depressive disorders, anxiety disorders, trauma-related disorders, and obsessive-compulsive disorders [15, 17].

Cognitive behavioral therapy (CBT) has been shown to be an efficacious treatment for emotional disorders [18, 19], and is recommended by treatment guidelines as a high-intensity treatments for depressive and anxiety disorders [20–28]. Most CBT protocols have been developed to treat one specific disorder. Recently, however, the accumulated evidence of shared psychopathology has influenced not only the conceptualization of emotional disorders, but also their treatment. Transdiagnostic psychological treatment has been developed to treat depression, anxiety, and trauma-related, obsessive-compulsive, and other emotion-related disorders by targeting their shared psychopathology [11, 29, 30]. The transdiagnostic approach has several potential strengths over the disorder-specific treatment: applicability to diverse disorders with comorbidity, simplification of treatment models for diverse emotional disorders, and ease of learning for novice therapists [30, 31].

Though the transdiagnostic approach for emotional disorders has been proposed only relatively recently, uncontrolled/nonrandomized and randomized controlled trials (RCTs) have already been mounted to investigate its efficacy and feasibility. Transdiagnostic psychological treatments vary in format (individual, group, internet), theoretical orientation (transdiagnostic cognitive behavior therapy, transdiagnostic mindfulness- and acceptance-based treatments), strictness or flexibility of the application of the protocol to individual patients, and range of disorders covered (anxiety disorders only, both anxiety and depressive disorders). The latest meta-analysis has identified 47 clinical trials (31 RCTs) on the transdiagnostic approach, with a total of 3705 participants. That study reported the cumulative effect sizes of transdiagnostic CBT on self-report measures among patients with diagnosis of depressive and/or anxiety disorders, in comparison to control groups (waiting-list, supportive counseling, psychoeducation, treatment-as-usual, etc.): medium for anxiety (*n* = 24, *g* = .65, 95 % CI .51–.79), large for depression (*n* = 22, *g* = .80, 95 % CI .62–.98), and medium for quality of life (*n* = 13, *g* = .46, 95 % CI .34–.57) [32, 33].

Despite the promise of transdiagnostic treatment, there are several limitations to it in its current state. First, the evidence of its efficacy is still inconclusive, because most existing trials have had a high risk of bias and because there is significant heterogeneity between the trials [32, 33]. Newby et al. reported that only seven out of 31 RCTs were rated low risk for bias in all of five criteria (namely, random sequence, concealment, blinding of assessors, incomplete outcome data, selective reporting); and clinical trials for transdiagnostic treatment on anxiety and depression in comparison to control group(s) have been shown to have moderate heterogeneity (*I*^2^ = 51–83 %) [32, 33]. More importantly, the latest meta-analysis did not examine the results for clinician-rated measures, because only 13 out of 31 RCTs reported an independent assessor rating for a structured clinical interview, and the interviews used varied from trial to trial. Second, no RCT for transdiagnostic CBT has been conducted in non-Western countries, which means that transdiagnostic treatment cannot yet be regarded as a transcultural treatment [12]. Third, only eight out of 31 RCTs have included both depressive and anxiety disorders, which are the putative populations for transdiagnostic treatment, which means that, many trials included a sub-category of anxiety disorders only. It is necessary to include transdiagnostic clinical populations in clinical trials to examine the external validity (i.e., generalizability) of the findings. Fourth, few studies have examined the putative treatment mechanism that rationalizes the treatment as transdiagnostic (e.g., neuroticism, emotion regulation) [12, 34]. Fifth, no study has examined the neuropsychological underpinnings of transdiagnostic treatment.

### Unified protocol for the transdiagnostic treatment of emotional disorders (UP)

The UP is one of the most empirically well-supported transdiagnostic psychological treatments [33, 35]. It was developed based on the recognition of diagnostic overlap across emotional disorders (i.e., comorbidity), non-specificity of treatment response to comorbid symptoms by diagnosis-specific treatments, latent structure of emotional disorders (e.g., negative affectivity), etiology, and commonality of findings of affective neuroscience on emotional disorders (e.g., reductions in connectivity between the amygdala and ventromedial prefrontal cortex) [11, 13]. Evidence relating to individual UP has been reported in various forms: theoretical paper [11, 13], case report [36], pilot clinical trial [37], and RCT with waiting-list [38]. Moreover, the feasibility of UP has been extended to individual and group formats for depressive and anxiety disorders [39], individual format for bipolar disorder [40], borderline personality disorder [41], and emotional disorders in adolescents [42].

The existing evidence, however, should be regarded as preliminary at best [12]. Findings by RCT to date are limited to one trial each for individual- and group-format UP, with small sample sizes [38, 39]. Further, there is clear lack of evidence regarding UP efficacy for depressive disorders and for Asian populations. Previous pilot studies for individual UP have examined only three patients with the principal diagnosis of major depressive disorder [36–38], and there is no RCT of UP efficacy for depressive disorders. Comparative clinical trials have been limited to one in the U.S. and one in Brazil [39].

Thus, we aimed to examine the efficacy of UP for Japanese patients with either or both depressive and anxiety disorders. Though such a broad category of participants constitutes a heterogeneous clinical population in terms of the standard diagnostic criteria (i.e., DSM-5 or *International Classification of Diseases* 10th revision; ICD-10) and leads to some methodological challenges unique to transdiagnostic treatment [43], we nevertheless selected this population because the unified protocol was originally developed to treat heterogeneous clinical populations transdiagnostically.

### Primary hypothesis and objectives

We hypothesize that the addition of the unified protocol to patients’ usual treatment by psychiatrists would be more efficacious in comparison to waiting-list for UP with usual treatment for the reduction of emotional symptoms among patients with depressive and anxiety disorders in a Japanese outpatient clinical setting.

### Treatment mechanism and process for enhancing the effectiveness of implementation of unified protocol

Though this clinical trial is mounted primarily to test the efficacy of the unified protocol on the primary outcome (i.e., emotional symptoms), it is also important to examine the treatment mechanism, process, and neurological correlates [12, 32]. Furthermore, because the UP was developed based on the findings on the shared underlying process of psychopathology across a wide range of emotional disorders (depressive, anxiety, trauma-related, and obsessive-compulsive disorders), it is important to examine how this shared process actually relates to the outcome of the treatment. These examinations are expected to not only allow theoretical refinement of UP, but also sharpen its clinical implementation. We focus on four different variables to examine the treatment mechanism; neuroticism, emotion regulation, emotion exposure, and anxiety sensitivity. These variables are considered to be transdiagnostic mediators/moderators of the treatment. Neuroticism here refers to the shared aspects of temperament commonly observed between depressive and anxiety disorders [44, 45]. Deficits of emotion regulation are also a putative transdiagnostic treatment mechanism of CBT [19, 34, 46]: through UP, patients will learn to engage adaptive emotion regulation and disengage maladaptive emotion regulation. The core module of the unified protocol is emotion exposure [35]; theoretically, the other modules will be expected to function to maximize the therapeutic effect of emotion exposure [11], and hence the patient’s ability to expose themselves to their own emotion should be one of the key focuses in terms of treatment mechanisms [47, 48]. Finally, anxiety sensitivity is regarded as the transdiagnostic process affecting the (non) maintenance of emotional disorders [49].

The effects of psychotherapy depend on its process: who delivers the protocol, how it is delivered, the degree to which the patient understands the concept or practices the skill, etc. [47, 50, 51]. Past studies suggest that strict adherence to CBT protocols does not linearly predict the treatment outcome [52, 53]. Hence, it is not enough to monitor adherence to the treatment protocol; one must also assess various aspects of the implementation or processes of the psychotherapy. In this clinical trial, we will collect some of the most important process measures regarding the implementation of the unified protocol. These include treatment expectancy [54, 55], therapeutic relationship [56–58], homework compliance [47, 59–61], and patient comprehension of treatment rationale. Examination of the relationships between these process measures and treatment outcome is expected to inform and help optimize the implementation of UP for patients with emotional disorders.

### Neurological correlates regarding the unified protocol

To the best of our knowledge, no study has yet examined neurological change after the intervention of transdiagnostic treatment; nevertheless, the existence of transdiagnostic neurological commonalities across emotional disorders has been suggested. Some researchers have developed the transdiagnostic neural model [62, 63] to reflect this. Recent studies have been focused more on the similarity of neural systems between depression and anxiety disorders, in addition to the differences [64]; this focus is consistent with the view taken by the Research Domain Criteria project of the U.S. National Institute of Mental Health [65]. In fact, neurobiological studies have shown some similarity in changes from before to after CBT across emotional disorders [66]. Considering that UP is a transdiagnostic treatment partly based on findings from the field of affective neuroscience [13], transdiagnostic change at the neurological level is indeed expected across emotional disorders. Moreover, recent investigation of the domain of neuropsychological perspective has predicted treatment efficacy using neurological measures; for example, Ball et al. [67] showed that the random forest model using pre-treatment neuroimaging data predicted treatment response of generalized anxiety disorder and panic disorder patients with good test characteristics. Hence, we will correct these neuropsychological data as an ancillary study.

## Methods/design

### Trial design, randomization, and ethical aspects

**Fig. 1:**
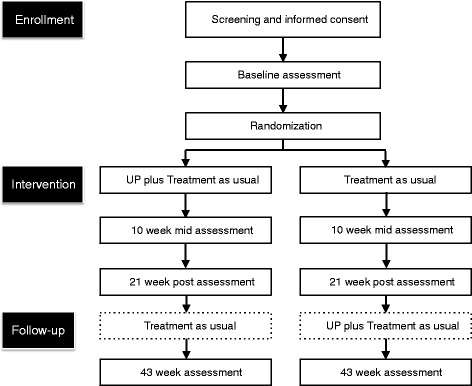
CONSORT diagram for this clinical trial

This study is a single-center, assessor-blinded, randomized, parallel-group superiority design with a target sample of 54 patients with depressive or anxiety disorders. Participant flow is depicted in Fig. 1. We will employ central randomization using Allocation and Registration Control System (ARCS) computer software, set up and managed independently of the study by the Project Management Office at the Keio Center for Clinical Research. A random sequence will be generated using minimization, with a ratio of 1:1 to balance stratified factors (depressive vs. anxiety disorder). Allocation will be implemented by the primary investigator or research coordinator via internet using a laptop in front of the eligible participant. This protocol has been reviewed and approved by the institutional review board of the (Japanese) National Center of Neurology and Psychiatry (accepted on October 18, 2013; A2013-092). All participants will give written informed consent to participation in the study. This trial is registered within ClinicalTrials.gov, number NCT02003261.

### Study setting

This single-site study has been conducted at the National Center of Neurology and Psychiatry (NCNP) Hospital in Tokyo; this is the national hospital specializing in the research and treatment of psychiatric, neurological, muscular, and developmental diseases in Japan. The hospital provides mainly secondary or tertiary inpatient and outpatient psychiatric treatments to socioeconomically diverse Japanese populations. Most new patients are referred to the NCNP Hospital because of non-response on their part to their usual care in their local clinic or hospital. Participants eligible for our pilot study had a long history from the first onset of the disorder (*Mean months* = 93.64, *SD* = 80.7) [68]. Treatment for depressive and anxiety disorders, usually provided in the NCNP Hospital outpatient department, had largely consisted of pharmacotherapy, supportive therapy, or active monitoring. Totals of 1017 and 2138 CBT sessions were respectively conducted in fiscal 2013 and 2014 at the hospital. In the Japanese medical setting, all patients basically continue their usual treatment with their treating psychiatrists when receiving any psychosocial treatments at the same medical institute. In the other words, the psychologist is not able to conduct any intervention with patients without the permission or direction of the treating psychiatrist. Along with the need for medical monitoring and clinical management, this is why all participants in both intervention and control groups need to continue treatment-as-usual (TAU) even if they are not pharmacologically prescribed. Japan’s universal health insurance system allows every Japanese resident to have access to any medical services at any medical institution nationwide without a gatekeeper and helps them pay incurred medical costs.

### Eligibility criteria

Participants need to fulfill all of the following inclusion criteria and to not meet any of the following exclusion criteria. We used DSM-IV criteria because the Japanese version of the DSM-5 had not yet been finalized and there was no structured method for diagnosing DSM-5 criteria (e.g., SCID-5) at the start of this trial.

#### Inclusion criteria

1. DSM-IV diagnosis of Major Depressive Disorder, Dysthymia, Depressive Disorder Not Otherwise Specified, Panic Disorder With Agoraphobia, Panic Disorder Without Agoraphobia, Agoraphobia Without History of Panic Disorder, Social Phobia (Social Anxiety Disorder), Obsessive-Compulsive Disorder, Posttraumatic Stress Disorder, Generalized Anxiety Disorder, Anxiety Disorder Not Otherwise Specified; as assessed by Structural Clinical Interview for DSM-IV-TR Axis I Disorders (SCID-IV-TR).
2. Depressive and anxiety symptoms are mild or severer (i.e., not absent) (GRID–Hamilton Rating Scale for Depression: GRID-HAMD > = 8).
3. Aged 20–65 years.
4. Gives full informed consent to participate in the study.

#### Exclusion criteria

1. No alcohol or substance use disorder in 6 months prior to the baseline assessed by SCID-IV-TR.
2. No current manic episode or current schizophrenia or other psychotic disorder at baseline assessed by SCID-IV-TR.
3. No serious suicidal ideation at baseline (GRID-HAMD Item 3 symptom intensity is less than moderate).
4. No life-threatening, severe, or unstable physical disorders or major cognitive deficits at baseline.
5. No evidence of inability to participate in half or more of the intervention phase.
6. No structured psychotherapy at baseline.
7. Other relevant reason as determined by the investigators.

### Intervention

We compare the UP with TAU intervention group with the waiting-list with TAU control group.

#### UP

UP is an transdiagnostic cognitive behavior therapy for emotional disorders [35]. Complete implementation usually takes 12–18 weekly sessions of 50–60 min each. Patient use the treatment workbook throughout the treatment [69], and practice newly learned cognitive-behavioral skills in between sessions. In this study, patients will receive approximately 16 face-to-face, individual sessions (range: 9–20) within 20 weeks. The therapist can shorten or lengthen needed sessions or intervals (weekly or biweekly) in accordance with the patient’s level of understanding or acquisition of the skills. Up to five additional sessions during the follow-up period for the intervention group will be allowed if the therapist and patient deem it necessary and if the patient has not completed all modules.

The unified protocol consists of one introductory session and eight modules for learning transdiagnostic cognitive behavioral skills: motivation enhancement, psychoeducation of emotion, emotion awareness training, cognitive reappraisal, emotion avoidance and emotion-driven behavior, tolerance training for physical sensations, emotion exposure, and relapse prevention (Table 1). Details of this intervention are described in the therapist’s guide to UP [35]. To be eligible for this clinical trial, a therapist needs to be qualified as a clinical psychologist by the Foundation of the Japanese Certification Board for Clinical Psychologists or possess a Japanese physician’s license, to have received adequate training using the therapist’s guide for UP and the CBT treatment manual published by the Japanese Ministry of Health, Labour and Welfare (at least 10 h of workshops), and to sit in on or listen to the full treatment course of at least two UP cases. At the beginning of this trial, four therapists (two females; clinical practice after certification of 4–6 years; experience of UP cases, 3–8 cases) were registered as staff therapists.

**Table 1:** Outline of Unified Protocol

Numbers of sessions needed	Module
1	Introduction to treatment
1–2	Motivation enhancement
1–2	Psychoeducation of emotional experiences
1–2	Emotion awareness training^a^
1–2	Cognitive appraisal and reappraisal^a^
1–2	Emotion avoidance and emotion-driven behaviors^a^
1	Awareness and tolerance training for physical sensations^a^
4–6	Emotion exposure^a^
1	Relapse prevention

^a^ Core modules

**Table 2:** Schematic diagram for the schedule of assessments

Time points (week)	0	1	2	3–4	5	6–9	10	11–14	15	16–20	21	43
Outcomes (evaluator rating)												
GRID-Hamilton Rating Scale for Depression	IE						IE				IE	IE
Structural Interview Guide for Hamilton Anxiety Rating Scale	IE						IE				IE	IE
Clinical Global Impression	IE						IE				IE	IE
General Assessment of Functioning	IE						IE				IE	IE
Structural clinical interview for DSM	IE										IE	IE
Outcomes (self-report)												
Overall Anxiety Severity and Impairment Scale	P	P	P	P	P	P	P	P	P	P	P	P
Overall Depression Severity and Impairment Scale	P	P	P	P	P	P	P	P	P	P	P	P
Disorder Specific Measures	P						P				P	P
EQ-5D	P						P				P	P
Sheehan Disability Scale	P						P				P	P
Sense of Authenticity Scale	P						P				P	P
Treatment mechanism												
Eysenck Personality Questionnaire Revised-Short version, Neuroticism subscale	P				P		P		P		P	P
Anxiety Sensitivity Inventory-III	P				P		P		P		P	P
Emotion Regulation Skills Questionnaire	P				P		P		P		P	P
Emotion Exposure Questionnaire	P				P		P		P		P	P
Treatment Rationale for Unified Protocol	P				P		P		P		P	P
Process measures												
Session Rating Scale		P/T			P/T		P/T		P/T			
Credibility/Expectancy Questionnaire			P/T									
Homework Compliance Scale			T	T	T	T	T	T	T	T		
Adverse Event			T	T	T	T	T/IE	T	T	T	IE	IE
Neurological correlates												
Functional Magnetic Resonance Imaging	X										X	X

*IE* assessment of independent evaluator, *P* patient self-report, *T* therapist self-report

Treatment adherence will be monitored by weekly group supervision and assessed using the Treatment Adherence Scale for UP. Two clinical psychologists (MH, MI), who translated the UP workbook and therapist guide, will supervise the study therapist. MH has clinical experience of more than 20 years. (MI will also serve as a staff therapist.) The Treatment Adherence Scale was developed by the developer of the unified protocol, and consists of 74 adherence items (12 for initial session, four for motivation enhancement, nine for psychoeducation, eight for emotion awareness training, eight for cognitive reappraisal, 11 for emotion avoidance and emotion-driven behavior, seven for tolerance of physical sensations, eight for emotion exposure, seven for relapse prevention). Items are rated according to whether the intervention has been implemented or not (Yes or No). To assess treatment adherence, we randomly select one-fourth of each participant’s sessions. The simple random sampling procedure was conducted using the True Randomization process (www.random.org) before the beginning of this clinical trial. The evaluation was conducted by a therapist who did not serve as a staff therapist for this study.

#### TAU

Though treatments for depressive and anxiety disorders in Japanese medical settings have not been systematically investigated, the majority seem to involve pharmacotherapy, unsystematic supportive therapy, or clinical monitoring by psychiatrists. The Japanese Society of Mood Disorders has published treatment guidelines for Major Depressive Disorders (MDDs) that recommend pharmacotherapy for moderate or severe MDD patients and pharmacotherapy and psychotherapy for mild MDD patients [70]. However, there are no such treatment guidelines for anxiety disorders specific to Japanese clinicians. One study showed that Japanese psychiatrists tend to choose pharmacotherapy for the first and second instance of treatment of MDD [71], and the results of our pilot study showed that almost all participants had received pharmacotherapy [68]. Based on the knowledge, we expected TAU for depressive and anxiety symptoms to be mostly pharmacotherapy. For this clinical trial, we defined TAU as any pharmacological or (non-systematic) psychological intervention except for systematic psychotherapy (e.g., any other type of cognitive behavioral therapy) and electroconvulsive therapy. We considered that the clinical success of participant’s treatment should be prioritized over our research purpose. Hence, we will not restrict any usual treatment by treating psychiatrists, including change of dose or type of drug. If treating psychiatrists start other systematic psychotherapy (e.g., another type of cognitive behavioral therapy) or electroconvulsive therapy, the patient will be excluded from the intervention in this study. Content of TAU will be documented in case report form.

### Follow-up phase

We set the assessment period at 43 weeks in order to exploratorily examine the trajectory after treatment for the intervention group. Participants assigned to the control group will receive the unified protocol in addition to TAU during the follow-up period (21–43 weeks). Data including the 43-week assessment will be used to exploratorily examine the persistence of the effect for the intervention group as well as the immediate treatment effect for the control group. There will be no treatment restrictions on the intervention group in the follow-up period.

### Discontinuation

Any participant in the intervention group who meets any of the discontinuation criteria will have their intervention (UP) stopped. Where possible, subsequent assessment will be conducted.

1. Participant request for discontinuation or participant withdrawal of consent.
2. Inability to contact the participant for a month.
3. Difficulty continuing the intervention because of a severe adverse event.
4. Difficulty continuing the intervention because of the exacerbation of comorbid symptoms.
5. Participant proves after assignment not to fulfill the eligibility criteria.
6. The whole clinical trial is stopped.
7. Any other reason that the primary investigator, therapist, and supervisor agree to warrants discontinuation.

### Measures

We will assess several measures of outcome, treatment mechanism, process, and neurological correlates. Most assessments will be conducted at baseline (0 weeks), 10 weeks (mid assessment), 21 weeks (post assessment), and 43 weeks (follow-up assessment). Table 2 shows the list and schedule of assessments.

#### Primary outcome

The primary outcome is change of depressive symptoms over the intervention period as assessed by the 17-item version of the semi-structured interview included in the GRID–Hamilton Depression Rating Scale (GRID-HAMD) [72–74]. GRID-HAMD employs a “grid” scoring system using the dimensions of symptom intensity and symptom frequency; higher scores indicate severer depressive symptoms (range: 0–52). This interview instrument has been reported to have excellent interrater reliability among Japanese clinicians (intraclass correlation coefficient; ICC = 0.95–0.99) [73], a finding borne out by our pilot study (ICC = .974, 95%CI = .966–.980) [68]. Internal consistency was reported to be 0.78 [74]. The interview instrument’s concurrent validity was demonstrated by high correlation between item and total score per the structured interview guide for HAMD [74]. We decided to use GRID-HAMD to assess “emotional symptoms” because it assesses not only depressive symptoms (depressed mood, guilt, suicide, insomnia early, insomnia middle, insomnia late, work and activities, psychomotor retardation, psychomotor agitation, loss of appetite, loss of sexual interest, loss of weight, and loss of insight) but also anxiety symptoms (psychic and somatic anxiety, general somatic symptoms, and hypochondriasis). Moreover, the GRID-HAMD is an appropriate choice for our study because it is one of the few depression rating measures that has been validated in Japanese and utilized in many clinical trials in Japan [75, 76], making implementation of assessment more straightforward. In our pilot study, we used GRID-HAMD and the Structured Interviews Guide for Hamilton Rating Scale for Anxiety (SIGH-A) as outcome measures. The result showed the GRID-HAMD showed higher treatment responsiveness than SIGH-A [68].

#### Secondary outcomes

Secondary outcomes for treatment efficacy are severity of anxiety assessed by SIGH-A, overall severity and improvement rated by Clinical Global Impression, responder status defined by 50 % or more baseline reduction in score on GRID-HAMD, remission of symptom defined as less than 8 on GRID-HAMD [77], and absence of met psychiatric diagnoses as assessed by SCID-IV-TR at baseline. These secondary measures are assessed at post-treatment (21 weeks).

#### SIGH-A

A semi-structured interview, SIGH-A includes 14 items for anxiety symptoms [78, 79]. Each anxiety-related item is rated from 0 to 4 on the basis of frequency, degree of distress, and symptom-related functional impairment during the previous week. Higher scores indicate more severe symptoms of anxiety (range: 0–56). Test–retest reliability and interrater reliability were respectively reported as 0.89 (95 % CI 0.83–0.93) and 0.99 (95 % CI 0.98–0.99). Concurrent and construct validity were demonstrated by high correlation with the traditional-format Hamilton Anxiety Rating Scale (*r* = 0.75–0.77) and equivalent correlations with self-report measures in the traditional format [78].

#### Clinical Global Impression (CGI)

CGI is an overall clinician rating of the severity of psychopathology (CGI-Severity; CGI-S) and improvement of it (CGI-Improvement, CGI-I) [80]. These are rated from 0 to 7 based on the assessor’s overall evaluation, which incorporates all available information about a patient’s history, psychosocial circumstances, symptoms, behavior, and the impact of symptoms on the patient’s ability to function [81]. In this study, essentially, the independent evaluator assesses the same patient at baseline, mid, post, and follow-up (0, 10, 21, and 43 weeks). Hence, the evaluator’s overall impression of longitudinal change will be reflected in the CGI-I rating.

#### Global Assessment of Functioning (GAF)

The GAF is a single measure for evaluating degree of global functioning, which is used for Axis V evaluation in DSM-IV [82]. A score is assigned from 0–100, based on symptom severity and the effects on social, occupational, and/or educational functioning.

#### Structural clinical interview for DSM-IV-TR axis I disorders (SCID-IV-TR)

SCID-IV-TR is a semi-structured interview used to asses the diagnostic status of DSM-IV-TR Axis I disorders [83]. We will administer modules A (Mood Episodes), B (Psychotic Symptoms), D (Mood Disorders), E (Substance Use Disorders), and F (Anxiety Disorders) at baseline to evaluate patients according to the inclusion and exclusion criteria. At post-intervention and follow-up, the independent evaluator administers part of each module to examine the presence or absence of the diagnoses met at baseline assessment. The Japanese version of *SCID-IV-TR* has been utilized in many clinical trials since its publication.

#### Overall Anxiety Severity and Impairment Scale (OASIS)

OASIS assesses the symptom of global anxiety by severity and impairment, using a (five-point) Likert scale [84, 85]. Items cover anxiety frequency, severity, and influence on symptoms. OASIS’s reliability and validity have been reported among U.S. and Japanese clinical and non-clinical populations [68, 84].

#### Overall Depression Severity and Impairment Scale (ODSIS)

ODSIS assesses the symptom of depression in terms of severity and impairment [86], on a (five-point) Likert scale. Similarly to OASIS, this scale assesses depression frequency, severity, and related impairment. Two studies suggest its reliability and validity among U.S. and Japanese clinical and non-clinical populations, respectively [86, 87].

#### Disorder-specific measures

In accordance with the diagnosis by SCID-IV-TR at baseline, each patient conducted the self-report measures to assess symptoms of met diagnoses. We used the Beck Depression Inventory-II (BDI-II) for Depressive Disorders [88, 89], Panic Disorder Severity Scale (PDSS) for Panic Disorder with or without Agoraphobia [90], Liebowitz Social Anxiety Scale (LSAS) for Social Anxiety Disorder [91], Penn State Worry Questionnaire (PSWQ) for Generalized Anxiety Disorder [92], Yale–Brown Obsessive Compulsive Scale (Y-BOCS) for Obsessive Compulsive Disorder [93], Impact of Event Scale-Revised (IES-R) for Posttraumatic Stress Disorder [94, 95], and Fear Questionnaire (FQ) for Agoraphobia without history of Panic Disorder [96]. All scales except for FQ have had their reliability and validity tested.

#### Euro Qol (EQ-5D-3L)

Patient quality of life is assessed by Euro Qol (EQ-5D-3L) [97, 98]. This scale consists of five items in the domain of quality of life (mobility, self-care, usual activities, pain/discomfort, and anxiety/depression) and one visual analogue scale regarding overall health status, ranging from 0 (worst imaginable health state) to 100 (best imaginable health state). Quality-adjusted life years will be calculated using the Japanese EQ-5D tariff [98].

#### Sheehan Disability Scale (SDS)

SDS is used to assess overall functional impairment [99, 100]. This scale consists of three domains of function (work/school, social life, and family life/home responsibilities). These items are rated on a 10-point visual analogue scale (from “not at all” to “extremely”). Its reliability and validity for a Japanese population has been reported [100].

#### Sense of Authenticity Scale (SOA)

SOA assess one’s sense that one is being true to oneself. It comprises seven items to be answered on a (five-point) Likert scale (1: Strongly disagree–5: Strongly agree). This scale was originally developed among a Japanese population [101]. Research in personality, social psychology, and positive psychology has suggested that authenticity can be conceptualized as a healthy type of self-esteem [101, 102] and can serve as an indicator of positive functioning [103]. Its reliability and validity has been repeatedly reported among Japanese population [101, 104].

#### Treatment mechanism

##### Neuroticism subscale of the Eysenck Personality Questionnaire Revised–Short-Form (EPQR-S)

Neuroticism is the tendency to experience negative affect frequently and intensely, which is a putative transdiagnostic factor for persistence of emotional disorders. This scale assesses this personality tendency using 12 dichotomous items. Its reliability and validity has been shown in the U.S. and in Japanese [105, 106].

##### Anxiety Sensitivity Index-3 (ASI-3)

ASI-3 is a self-report questionnaire for assessing beliefs about the feared consequences of symptoms associated with harmful physical, cognitive, or social concerns [107]. The questionnaire consists of 18 items evaluated on a four-point Likert-type scale (0: very little; 4: very much). The measure possesses excellent psychometric properties, performing well on various indices of reliability and validity [107].

##### Emotion Regulation Skills Questionnaire (ERSQ)

ERSQ assesses how the respondent has dealt with negative emotions in the previous week [108]. This 27-item questionnaire has nine subscales: awareness, clarity, sensation, understanding, compassionate self-support, modification, acceptance, tolerance, and readiness to confront. Each subscale comprises three items, measured on a (five-point) Likert scale (0: not at all; 4: almost always). The scale demonstrates sufficient reliability and validity [108].

##### Emotion Exposure Questionnaire (EEQ)

EEQ was originally developed to assess overall patient tendency to expose themselves to their own emotions, which is understood to be a hallmark of the treatment mechanism of the unified protocol. The items represent willingness or ability to expose oneself to emotions (e.g., “Even if it is distressing, I try to feel the emotion fully”). This questionnaire consists of thirty items evaluated on a (five-point) Likert-type scale.

##### Treatment Rationale of Unified Protocol (TRUP)

TRUP was originally developed to assess comprehension of treatment rationale for the unified protocol. It consists of 12 statements to be answered yes or no. We developed two items for each treatment module (psychoeducation of emotion, emotional awareness, cognitive reappraisal, emotion avoidance and emotion-driven behavior, tolerance of physical sensation, and emotion exposure). A sample item regarding psychoeducation of emotion is “It is better to suppress emotion and feelings and try not to feel it.”

#### Process measures

##### Session Rating Scale (SRS)

SRS is used to assess the therapeutic alliance [109]. It consists of four visual-analogue items (bond, consensus of task, consensus of goal, and overall evaluation). These item are consistent with the conceptualization of the “working alliance” and have good psychometric properties in terms of reliability and validity [109].

##### Credibility/Expectancy Questionnaire (CEQ)

CEQ assesses the patient’s perception of the credibility of treatment and expectancy regarding treatment. It measures cognitive and emotional aspects of credibility and expectancy, using six items. Because there was no Japanese version of this questionnaire, we translated it using a rigorous back-translation procedure, with the permission and support of the original developer of this questionnaire.

##### Homework Compliance Scale (HCS)

HCS is a single measure for the therapist to assess the patient’s degree of completion of their homework [110]. Therapists who conduct the unified protocol assess patient homework compliance at the end of every session, using 0–6 anchors. We translated this measure using a back-translation procedure, with permission and support from one of the original authors of the scale.

#### Adverse events, treatment, and assessment integrity

We defined adverse events to include any undesirable physical or psychological event during the research period, irrespective of its relation with the intervention. Using the forms described above, therapists or independent evaluators solicit patients at each visit for assessment of occurrence or worsening of the following symptoms: dry mouth, astriction, dysuria, accommodation disorder, orthostatic hypotension (dizziness, lightheadedness), sleepiness, malaise, insomnia, anxiety/irritation, asitia, weight gain or loss, loss of sexual interest, suicidality, suicidal attempt, and other symptoms. We assessed the severity of the adverse event using criteria from the Japanese version of the Common Terminology Criteria for Adverse Events (CTCAE v4.0) [111]. This rating system uses a (five-point) Likert-type scale (1: Grade 1, Mild; 2: Grade 2, Moderate; 3: Grade 3, Severe or medically significant but not immediately life-threatening; 4: Grade 4, Life-threatening consequences; 5: Grade 5, Death). A “severe” adverse event is defined as one with a 3 or higher severity rating.

#### Neuropsychological assessment

Data are acquired using a 3-tesla Siemens Verio magnetic resonance imaging (MRI) scanner (Erlangen, Germany) with a standard Siemens 32-channel phased array head coil. The MRI protocol includes acquisition of (1) resting-state functional MRI (7 min), (2) T1-weighted imaging (4 min), (3) fluid attenuated IR (FLAIR) (4 min), (4) diffusion weighted imaging (DWI) (8 min), for a total of under 23 min of scanning. Resting-state fMRI will be conducted to measure functional region connectivity, and DWI to measure anatomical region connectivity. T1-weighted images and FRAIR images will be used for the volumetric analysis. Participants who meet any exclusion criteria for MRI (e.g., currently pregnant; currently breastfeeding; wearing pacemaker, aneurysm clips, or other implants) will be excluded from the MRI scan.

### Procedure for assessment; blinding

Independent evaluators not involved with the intervention for or coordination of this clinical trial will conduct the SCID-IV-TR, GRID-HAMD, SIGH-A, and rate CGI and GAF. These independent evaluators are prohibited from accessing any information that could confer participant assignment. Independent evaluators need to be trained at least 20 h to be eligible to serve. For the primary outcome of GRID-HAMD, the evaluators trained by attending workshops, watching a training DVD, role-play with a mock patient, direct observation of evaluation, and conducting GRID-HAMD with patients. All evaluator assessments will be recorded and used for other evaluator ratings to examine the reliability of the assessment. We will choose one-fifth of the assessments using the same random sampling technique as for treatment adherence, and examine assessment integrity on their basis.

The independent evaluators will respond to the modified version of the Independent Evaluator Knowledge of Treatment scale at mid (10 weeks), post (21 weeks) and follow-up assessment (43 weeks). This three-item scale for assessing the evaluator’s knowledge of patient assignment has been used in clinical trials of cognitive behavior therapy for panic disorder as well as the unified protocol for emotional disorders [112]. The independent evaluators give their conjectures, rate their confidence in their answer, and provide any relevant information that led to the conjecture.

### Data management and monitoring

Acquired data will be entered immediately into a database constructed using Microsoft Access. Each entry form will be restricted to the possible range of items. Data entry and verification will be conducted independently by study assistants. Central and onsite monitoring will be conducted periodically by a data manager at NCNP throughout the course of the trial.

### Sample size calculation

Because this clinical trial is intended to examine the efficacy of a novel treatment (the unified protocol) that targets clinical populations that are heterogeneous in terms of a traditional diagnostic perspective, we had to collect information on the treatment effect size from various sources in order to estimate the sample size. Estimation was based on information from previous findings on efficacy of transdiagnostic and disorder-specific CBT for depressive and anxiety disorders, results of our pilot study, and previous randomized controlled trials of the unified protocol among U.S. participants [38, 68].

Meta-analysis of the RCT to examine the effect of CBT in comparison to waiting-list on major depressive disorder reported a standardized mean difference (SMD) of 0.82–0.85 [113, 114]. For anxiety disorders by DSM-IV criteria, separate meta-analysis of RCT comparing CBT with waiting-list or seemingly equivalent condition (e.g., no treatment, or placebo attention control) showed an effect size of 0.82 for generalized anxiety disorder (*n* = 4) [115], 0.91 for panic disorder with/without agoraphobia (*n* = 7) [116], 0.93 for social anxiety disorder (*n* = 7) [117], and 1.36–1.70 for posttraumatic stress disorder (*n* = 21) [118]. Finally, other meta-analysis reported a large effect size of CBT against waiting-list or active placebo control for emotional disorders (SMD = 0.63–1.09) [119, 120].

There are few previous findings on the efficacy of the unified protocol. In the pilot randomized controlled trial comparing with waiting-list, an SMD of 0.52–1.11 for depression measures and 1.10 for anxiety measures was observed among patients with anxiety disorders [38]. Our pilot clinical trial of the unified protocol among Japanese patients with depressive and anxiety disorders demonstrated a within SMD of 1.70 (95 % CI = 0.81–2.67) for GRID-HAMD, 1.14 (95 % CI = 0.40–1.89) for ODSIS, 0.76 (95 % CI = −0.05–1.57) for BDI-II, 1.29 (95 % CI = 0.56–2.06) for SIGH-A, and 1.71 (95 % CI = 0.91–2.50) for OASIS [68].

Summarizing these findings, we assumed a large effect size of 0.85 for UP with TAU against waiting-list with TAU for the reduction of primary outcome assessed by GRID-HAMD. Using G*Power, in order to detect the difference between groups with a statistical power of 80 % for a two-tailed test, the needed sample size was calculated to be 23 for each group. In consideration of reported dropout rates of 11.76–15.39 % in previous studies [38, 68], we assumed a dropout rate of 15 % and a need to have at least 27 participants in each group to test the primary hypothesis of this study (difference of GRID-HAMD scores between intervention and control groups over the 21-week period). We will continue recruiting patients for ancillary study of neuroimaging, treatment process, and mechanism.

Two separate meta-analyses reported the effect size of transdiagnostic cognitive-behavioral treatment *after* we had finished designing this clinical trial and had started the actual trial [32, 33]. These latest analyses showed the effects of transdiagnostic treatments (including individual, group, and internet format) with control group (waiting-list, supportive counseling, psychoeducation, TAU, online support, or discussion forum) on the self-report measure of anxiety (SMD = .65, 95 % CI = .34–.57, *n* = 24) and depression (SMD = .80, 95 % CI = .62–.98, *n* = 22). Reinholt and Krogh [33] reported pooled SMD for four RCTs testing the efficacy of transdiagnostic individual or group treatment in comparison to waiting-list controls as −1.00 (95 % CI −1.74–-0.26). Taking into account this information, our estimation might have been a bit optimistic, suggesting the risk that our method is underpowered to test our primary hypothesis.

### Statistical methods

All analyses for testing efficacy of treatment on primary and secondary measures will be analyzed on the basis of the intent-to-treat principle, using a linear mixed model (LMM). The LMM approach was selected because of its strength in dealing with missing data and its ability to incorporate random effects (i.e., of the participants) into the analyses. For the primary outcome, the dependent variable is the GRID-HAMD score, and the independent variables are assignment (i.e., intervention vs. control group), time (i.e., 0, 10, and 21 weeks), and interaction of the assignment and time, as a fixed-effect variable, and participants, as random-effects variable. We will construct a conditional growth model [121] using a restricted maximum likelihood estimation method to compare changes in depression severity between groups from baseline to 21 weeks. The assessment period is measurement time for the growth model (weeks 0, 10, and 21), which will be coded as an ordinal scale (0, 1, 2). We assume an unstructured error covariance for this model. If the analysis will not converge, we will use other covariance structures. The covariance structure that will show convergence of the analysis and best fit to the data, evaluated by Akaike’s information criterion, will be used as the primary analysis.

To test robustness, we will conduct the same LMM, including a stratified variable (depressive vs. anxiety disorder) as covariate and other sensitive analyses taking into account the following aspects: ITT vs. per-protocol, and multiple imputation methods for missing data [122]. Secondary outcomes will be analyzed in the same way as primary outcomes. For all analyses, *P* < .05 will be considered statistically significant. The other statistical analyses will be conducted to examine treatment mechanism, process, and neurological implications in relation to outcome.

### Recruitment

This study will be announced to the psychiatrists at the National Center of Neurology and Psychiatry (NCNP) Hospital. When a patient requests cognitive behavioral treatment and their primary psychiatrist deems it feasible for that patient, the psychiatrist refers the patient to the hospital’s department of clinical psychology. After the intake session for CBT, the team in that department, consisting of psychiatrists and clinical psychologists, holds a conference to determine the most appropriate CBT program for that patient. Patients with depressive and anxiety disorders who seem suited to this study will be referred to this clinical trial.

## Discussion

Completion of this clinical trial will add to the evidence of efficacy for transdiagnostic psychological treatment of emotional disorders, which is not conclusive at this time [32, 33]. This trial has importance especially with regard to the question of the efficacy of UP for depressive disorders and in an Asian population. UP is considered to be promising as a treatment for depressive disorders because of the enormous efficacy shown in existing findings for treatment of this disorder by diagnosis-specific CBT [18, 113, 123]. There seems to be no reason to expect weaker efficacy with UP, and there is some reason to expect enhanced efficacy of UP on depressive disorders. That is, one of the core modules of UP is emotion exposure, and the rationale of “avoiding avoidance” was originally considered important for treatment of anxiety disorders; however, recent advances in the understanding of behavioral activation lead us to regard avoidance as a key maintenance factor for depression. Further, positive as well as negative emotion regulation has been found to be an important treatment target [124]. The UP has been modified to incorporate these findings in order to enhance its efficacy [37].

Transdiagnostic treatments have several strengths, including versatility to treat a broad range of emotional disorders, and the relative parsimony of learning core principles of cognitive behavioral therapy. These strengths are especially important to clinical practice in Japan, which has many barriers that hinder patients from receiving evidence-based treatments and where there are severe problems for the dissemination of CBT [125]. Only three randomized controlled clinical trials have been conducted to assess the efficacy of CBT in medical settings in Japan (one trial each for obsessive–compulsive disorder, insomnia, and hypochondria). If its efficacy is confirmed by the present study, this versatile treatment will contribute to the more rapid adoption of evidence-based treatment to those who need it for emotional difficulties in Japan.

In addition to the results for the primary hypothesis, secondary analyses of treatment mechanism, process, and neurological correlates will inform better implementation of the unified protocol, and, possibly, other transdiagnostic treatment.

### Current study status

The JUNP study began recruiting participants in December 2013. Forty-four participants have been enrolled at the time of submission of this protocol.

## Abbreviations

JUNP: Japan unified protocol clinical trial for depressive and anxiety disorders
DSM: diagnostic and statistical manual for mental disorders
UP: unified protocol for transdiagnostic treatment of emotional disorders
CBT: cognitive behavioral therapy
RCT: randomized controlled trial
ICD: international classification of diseases
NCNP: national center of neurology and psychiatry (Japan)
TAU: treatment-as-usual
SCID-IV-TR: structural clinical interview for DSM-IV-TR axis I disorders
GRID-HAMD: GRID-Hamilton rating scale for depression
MDD: major depressive disorders
ADIS-IV: anxiety disorder interview schedule for DSM-IV
SIGH-A: structured interviews guide for Hamilton rating scale for anxiety
CGI: clinical global impression
GAF: global assessment of functioning
OASIS: overall anxiety severity and impairment scale
ODSIS: overall depression severity and impairment scale
BDI-II: Beck depression inventory-II
PDSS: panic disorder severity scale
LSAS: Liebowitz social anxiety scale
PSWQ: Penn State worry questionnaire
Y-BOCS: Yale–Brown obsessive compulsive scale
IES-R: impact of event scale-revised
FQ: fear questionnaire
EQ-5D: euro Qol
SDS: Sheehan disability scale
SOA: sense of authenticity scale
EPRQ-S: Eysenck personality questionnaire revised–short-form
ASI-3: anxiety sensitivity index-3
ERSQ: emotion regulation skills questionnaire
EEQ: emotion exposure questionnaire
TRUP: treatment rationale of unified protocol
SRS: session rating scale
CEQ: credibility/expectancy questionnaire
HCS: homework compliance scale
CTCAE: common terminology criteria for adverse events
MRI: magnetic resonance imaging
LMM: linear mixed model
ARCS: allocation and registration control system
